# Prediction and Sensitivity Analysis of CO_2_ Capture by Amine Solvent Scrubbing Technique Based on BP Neural Network

**DOI:** 10.3389/fbioe.2022.907904

**Published:** 2022-06-20

**Authors:** Jiangtao Fu, Yufeng Chang, Bijie Huang

**Affiliations:** ^1^ State Key Lab of Precision Blasting, Jianghan University, Wuhan, China; ^2^ Hubei Key Lab of Blasting Engineering, Jianghan University, Wuhan, China; ^3^ Hubei (Wuhan) Institute of Explosion Science and Blasting Technology, Jianghan University, Wuhan, China; ^4^ School of Environment and Health, Jianghan University, Wuhan, China; ^5^ Hubei Key Laboratory of Industrial Fume and Dust Pollution Control, Jianghan University, Wuhan, China

**Keywords:** bionic algorithm, BP neural network, CO_2_ capture, sensitivity analysis, prediction accuracy

## Abstract

With the rapid development of artificial intelligence, bionic algorithm has been gradually applied in various fields, and neural network has become an important and hot issue in the field of scientific research and engineering in recent years. This article proposes a BP neural network model to predict the capture ability and sensitivity of CO_2_ in monoethanolamine (MEA) aqueous scrubbing technique from a 2 × 1,000 MW coal-fired power plant expansion project in eastern China. The predicted values agree well with the experimental data with a satisfactory mean square root error (MSRE) ranging from 0.001945 to 0.002372, when the change in the circulation amount of MEA and the accuracy of prediction results of the back propagation neural network (BPNN) algorithm is as high as 96.6%. The sensitivity analysis results suggested that the flue gas amount has a marginal effect on the system performance, while further attention should be paid to the MEA circulation amount, which is crucial to the CO_2_ capture amount. The temperature profiles show the typical behavior of the reactive absorption column where a temperature bulge can be seen at the bottom of the column due to the high L/G ratio of the experimental and prediction results. The coefficients of correlation *R* ^2^ with the change of MEA circulation amount, change of CO_2_ concentration, and steam consumption are 0.97722, 0.99801, and 0.98258, respectively. These results have demonstrated that the present study has established the BPNN algorithm as a consistent, reliable, and robust system identification tool for CO_2_ capture by the amine solvent scrubbing technique of operation in coal-fired power plants.

## 1 Introduction

Artificial neural network technology is made up of a large number of neurons. The whole network mainly includes three parts, namely, the input layer, hidden layer, and output layer. Neurons at each layer of the network are connected to the threshold through weights. The training process of the network is the process of constantly adjusting the weights between the layers according to errors under the condition of a given target input and target output. Artificial neural network has not only a powerful function in nonlinear data processing ability but also the advantages of self-organization, self-adaptation, and self-learning. Its method is simple, and strong operability can effectively predict the sensitivity analysis of CO_2_ capture efficiency of monoethanolamine (MEA) water scrubbing technology. BP neural network is a kind of multilayer feed-forward neural network which corrects errors by the error back propagation algorithm. Its core characteristic is such that the signal goes forward, and the error is the back propagation. In the process of forwarding propagation, the input signal passes through the input layer, the hidden layer is processed layer by layer, when it comes to the output layer. If the result does not meet the expectation, then it goes into the process of back propagation, and returns the error signal to modify the weight of each layer. Aiming at the problem of low accuracy of reliability prediction in CO_2_ capture, a back propagation neural network (BPNN) model is developed.

Carbon dioxide is the main emission during the coal-fired power generation process, which is a kind of greenhouse gas and the most important reason for causing global warming (Cui et al., 2018; Tapia et al., 2018). Carbon Capture, Utilization, and Storage (CCUS) technology demonstration projects were implemented worldwide (Fragkos, 2021), and it has become a major and hot topic in scientific research and engineering in recent years (Tapia et al., 2018; Ling et al., 2019; Khoshraftar and Ghaemi, 2022; Raidoo and Laubscher, 2022). MEA organic amine absorption, compression, and refinery are widely used as the technical path in fulfilling the CCUS targets (Ling et al., 2019; Zhang et al., 2017; Khoshraftar and Ghaemi, 2022). MEA-based carbon capture process is proven to be the most mature and economically appealing option (Wu et al., 2020).

The modeling and simulation of CO_2_ capture processes with amine solutions are considered important developments toward the detailed study and analysis of these processes (Afkhamipour and Mofarahi, 2014). Few previous studies have been published on full-scale CO_2_ capture projects, and most of them have involved very small sample sizes or pilot scales. Thus, a full-scale project application of modeling and simulation of CO_2_ capture processes must be considered. It is necessary to develop algorithms that are inexpensive and easy in predicting the efficiency and energy consumption for CO_2_ capture in a benchmark coal-fired power plant flue gas process. Bioinspired optimization is a growing research topic with many competitive algorithms being proposed every year and contains Evolutionary Computation and Swarm Intelligence (LaTorre et al., 2021). Particle Swarm Optimization (PSO) is an optimization bioinspired algorithm for its promising performance in many fields (Li et al., 2019). The artificial neural network (ANN) algorithm simulates the social behavior of agents that interact with each other by acting on their local environment (Alateeq and Pedrycz, 2021). In this article, the back propagation neural network (BPNN) is used as the algorithm to predict CO_2_ capture efficiency and sensitivity analysis based on the MEA aqueous scrubbing technique.

The absorption mechanism (Danckwerts, 1979) and regeneration mechanism (Zhang et al., 2008) is showed in Eq. 1 and Eq. 2

$$CO_{2}+2RR^{\prime }\mathrm{NH}↔RR^{\prime }\mathrm{NCO}O^{-}+RR^{\prime }NH_{2}^{+}$$ (1)

is the CO_2_ adsorption reaction by MEA, where R is alkyl group and R′ is H for the primary amines and alkyl for the secondary amines.

$$RR^{\prime }\mathrm{NCO}O^{-}+H_{2}O\overset{\mathrm{Heat}}{↔}CO_{2}+RR^{\prime }\mathrm{NH}+OH^{-}$$ (2)

is the regeneration reaction of MEA. From the reaction, Eq. 2, it can be seen that carbamate (RNHCOO−) transforms into amine and CO_2_. The enthalpy of dissociation for CO_2_ release depends on the stability of carbamate formation.

The key contributions of this work are

1) The present research status of CO_2_ capture capability and sensitivity prediction of CO_2_ in MEA water scrubbing technology were analyzed, and the shortcomings of the existing research were pointed out.
2) A prediction method of CO_2_ capture capability and sensitivity of MEA water scrubbing technology based on BPNN was proposed.
3) The prediction results of the proposed network model are compared with the experimental results to verify the effectiveness of the proposed method. It is critical to deduce the relationship between these results and the circulation amount of MEA, steam consumption in the regeneration tower, total flue gas flow, the ratio of liquid–gas (*L/G*), and the average molecular weight of lean liquid and heat required for the regeneration per unit of MEA, and the key influencing factors being analyzed and evaluated.

This article is organized as follows. In Section 2, related work comparing different algorithms and implementation is presented. In Section 3, the ProTreat software is used to simulate the overall process flow of the CO_2_ capture process and obtain the amount of CO_2_ capture data. The BP algorithm is used to build a neural network to train it. The key factors for CO_2_ capture and the MEA regeneration process are investigated. In Section 4, the results are presented along with the comparison and discussion of the experimental data and prediction value by the BP model. The network parameters are used to establish a corresponding mathematical prediction model and analyze the sensitivity of the capture process flow model. The conclusions are presented in Section 5.

## 2 Related Work

The ability of ANNs in modeling highly nonlinear systems lies in possessing nonlinear transfer functions and their capability to learn and recognize different patterns by adjusting their parameters, i.e., synaptic weights (w) and biases (b), during the training process. One of the advantages of ANN modeling is that there is no need for prior consideration of any functional relationship between the variables as it is common in proposing correlations (Sipocz et al., 2011; Nguyen et al., 2007). The input data are introduced to the network through the input layer and is then transferred into the hidden layers. Eventually, the network responses are stored in the output layer (Hagan et al., 2002). This type of neural network in which the data always flow in a forward direction is typically called the multilayer feed-forward ANN.

The current research based on solvent-based carbon capture lie primarily on steady optimization, which may consider the effect of different solvents, operation parameters, configurations, and techno-economic analysis (Mac Dowell and Shah, 2015; Van De Haar et al., 2017; Zhang et al., 2017; Bui et al., 2018). However, the steady-state models are unable to replicate the transient behavior of the actual carbon capture process and cannot provide precision information for controller designing. Dynamic modelling that is based on two different approaches, namely, the equilibrium approach and rate-based approach is therefore presented. Lawal et al. (2010) proved that the rate-based approach gives more accurate results in predicting the temperature profile in an absorber. Various simulation software have been used for modelling, including gCCS, gPROMS, Aspen Plus, MATLAB, and Modelica (Liao et al., 2018; Mac Dowell and Shah, 2015; Zhang et al., 2017; Arce et al., 2012). Artificial neural networks (ANNs) with nonlinear mapping capability have been successfully employed in modeling the VLE (Vapor-Liquid Equilibrium) data of various systems in chemical engineering (Lashkarbolooki et al., 2013; Zarenezhad and Aminian, 2011; Pahlavanzadeh et al., 2011). The ability of ANN to model the nonlinear processes allows its implementation for a wide range of diverse applications (Kim et al., 2017).

Chen et al. (2021a) developed a complex network dynamics model to predict the multidimensional results in the context of derived topics. Zheng et al. (2011) verified the feasibility of a hybrid genetic algorithm for the optimization and pattern search and put forward new challenges for the monitoring mode. Chen et al. (2022) predict the effects of the dual circulation promotion policy based on the system dynamics model and achieve an accurate output. Braik et al. (2020) used the hybrid neural network (NN) models to predict the PM and ozone concentrations. The proposed models in this study include recurrent multilayer perceptron (RMLP), recurrent fuzzy neural network (RFNN), and hybridization of grey wolf optimizer (GWO) and RFNN. Xu et al. (2022) propose a genetic-based model to optimize the operation results of the 3D Burch–Schneider cage. Evolutionary game of multi-subjects model was used to predict the live streaming results by Chen et al. (2021b). Liu et al. (2022) optimize the trajectory for digital twin robots by the genetic algorithm (GA). Guo et al. (2020) proposed an air pollution forecast model using a deep ensemble NN that combines the efficiency of GRU, LSTM, and recurrent neural networks (RNNs) to predict PM_2.5_ concentrations which is presented.

## 3 Materials and Methods

This study is based on a CO_2_ capture demonstration project in a 2 × 1,000 MW expansion project of a coal-fired power plant in eastern China, and the operation data were collected from the onsite engineering parameters.

### 3.1 Assumptions in ProTreat Modeling

In this study, a ProTreat method was used as the simulation tool to acquire the data. Rochelle (2009) proposed a set of different processing parameters in the simulation process. To simplify the simulation process, the following basic assumptions are made:

1) The main components of power plant flue gas contained only carbon dioxide, nitrogen, and oxygen.
2) There are no pressure drops in the operating units other than in the absorption tower and regeneration tower, which is zero during the simulation.
3) MEA is periodically recovered during CO_2_ capture, assuming the return flow is 10% of the total flow.
4) The MEA solution replenishment is 10% of the initial required solution volume, and the circulating water replenishment is 130 m^3^/h. In the process of CO_2_ capture, there are MEA and moisture loss.

### 3.2 Back Propagation Neural Network Algorithm Basics

**FIGURE 1:**
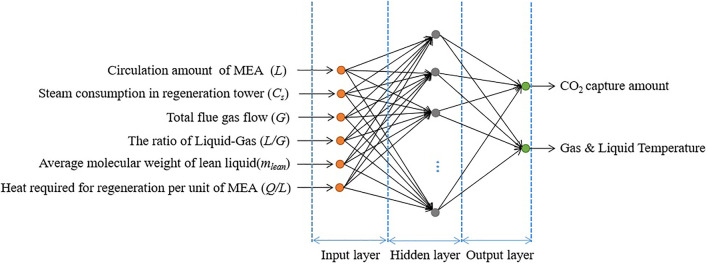
Structure of the BPNN for amine solvent scrubbing CO_2_ capture in coal-fired power plant.

In terms of structural network, a general BPNN consists of an input layer, hidden layer, and output layer. A fully established BPNN has been depicted in Figure 1. In this input layer, the number and structure of neurons have to be determined. The input neurons strongly depend on the related physical quantities and must be very sensitive to the predicted results. To some extent, different hidden layers with more neuron numbers should have a better potential for predictive performances. However, the long train time and local convergence are very apparent. In general, we can design the hidden structure from simple one layer to more complex layers, if necessary. There is no evidence that the structure with more hidden layers has an accurate prediction.

The BPNN prediction model is divided into two parts: one is the forward input of parameters information and the other is the reverse input of error. The input layer obtains the effective information from the outside and transmits it to the hidden layer in the middle. After the information is transformed, it is transmitted to the output layer to realize the forward propagation of the signal. If the output result cannot meet the expected condition, the error signal will be back propagated. The number is calculated in the reverse direction according to its connection method. The continuous input of information variables and negative information variables keeps the weights and thresholds in a state of dynamic adjustment by the error gradient descent method and finally obtains the results close to the expected values.

The BPNN with one hidden layer has the characteristic of infinite approximation to any nonlinear continuous function, so a three-layer forward-feed BPNN is constructed. The accomplishment of success of an ANN model highly depends on a clear understanding of the situation under study and the selection of the most significant input variables (Kakatia et al., 2019). This study considers the degree of influence of the control parameters on the response parameters. Six factors were chosen as the input neurons in the BPNN algorithm, namely,

1) circulation amount of MEA (*L*),
2) steam consumption in the regeneration tower (*C*_*s*_),
3) total flue gas flow (*G*),
4) the ratio of liquid–gas (*L/G*),
5) average molecular weight of lean liquid (*m*_*lean*_), and
6) heat required for the regeneration per unit of MEA (*Q/L*).

The structure of the BPNN is shown in Figure 1.

Neural network toolbox of MATLAB was employed to develop the ANN model specifying the number of layers and the training function. Network with the BPNN algorithm, which is very well suited to the training of the neural network, was used to construct the network architecture of the ANN and to evaluate the method convergence (Saini and Soni, 2002). In the BP algorithm, the responsibility for reducing the output error is shared among all of the connection weights. The network usually has one or more hidden layers where one hidden layer is normally adequate for modeling the nonlinear and complex functions. Thus, the proposed network consisted of one input layer with up to 6 neurons, one hidden layer with up to 12 neurons, and one output layer with up to 2 output neurons to obtain an accurate prediction, as shown in Figure 1.

The experimental data used in this simulation study of CO_2_ absorption using MEA solutions are presented in Table 1.

**TABLE 1:** The experimental conditions for the CO_2_ absorption.

Parameters	Units	Values
Total gas flow rate	m^3^/h	1,100,000
Liquid flow rate	m^3^/m^2^h	10.0
MEA concentration	kmol/m^3^	3.0
Inlet CO_2_ loading	mol CO_2_/mol amine	0.25
CO_2_ content in feed gas	mol%	10.0
CO_2_ removal efficiency	%	95
Liquid temperature	°C	42
Gas temperature	°C	28
Pressure	kPa	101.3

These six neurons are the typical factors that will influence the CO_2_ capture efficiency in the whole process. CO_2_ capture amount and the temperature of gas and liquid are the two output parameters. The basic processing unit of the neural network is the nonlinear input–output relationship and the sigmoid function is chosen as the transfer function of the BP neural network, see Eq. 3.

$$f\left(x\right)=\frac{1}{1+e^{-x}}.$$ (3)

**FIGURE 2:**
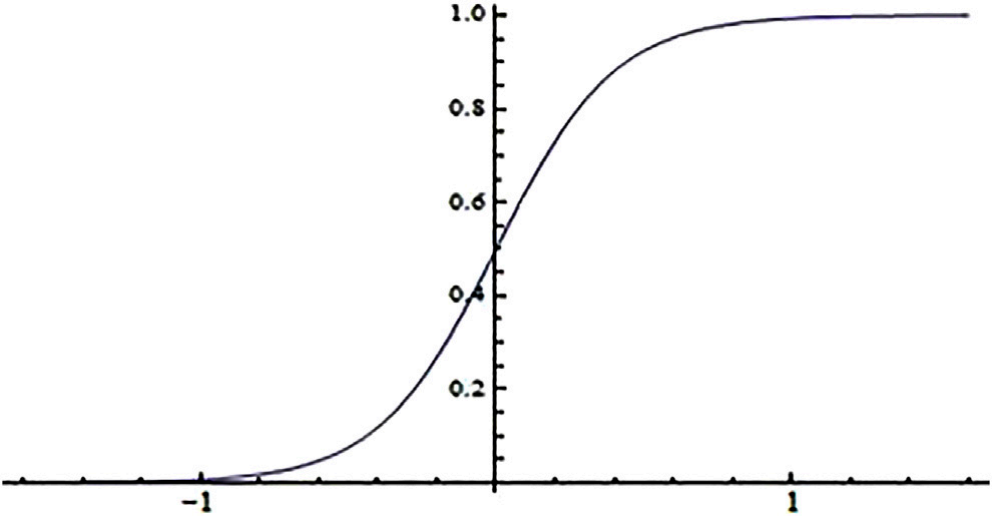
The graph of sigmoid function.

In the training process of the neural network, by constantly changing all the parameters in the neural network, the loss function is continuously reduced, thereby a higher-accuracy neural network model is proposed. The relationship between the input and output values in the sigmoid function is shown in Figure 2. According to the characteristics of the sigmoid function, it is necessary to convert the input quantity and output quantity between [0, 1]. A fully nonlinear relationship is established between the input and output, and Eq. 3 is the main form of neuron network representation.

To evaluate the prediction performance of the neural network, the mean square root error (MSRE) is adopted in this article, and the specific calculation formulas are as follows:

$$MSRE=\sqrt{\sum_{i=1}^{n}\frac{{\left(t_{i}-o_{i}\right)}^{2}}{n}},$$ (4)

where *t*_*i*_ is the observed value, *o*_*i*_ is the predicted value, and *n* is the number of samplings in the data set.

The activation function is the *tansig* function, and the training function is the *trainlm* function. In the input layer, *x* is the input data matrix, *IW* is the weight, and *a* is the threshold from the input layer to the hidden layer. In the output layer, y is the output data matrix, *LW* is the weight, and *b* is the threshold from the hidden layer to the output layer. The relationship can be present as in Eq. 5.

$$y=\frac{2^{*}LW}{1+e^{-2^{*}\left(IW^{*}x+a\right)}}-LW+b.$$ (5)

In the algorithm, *C*, *m*_*lean*_, *T*_*fg.in*_ *, L/G*, *Q/L*, and *y*_*CO2*_ were chosen as the input data, the output value of *y* can be calculated according to Eq. 5, and the algorithms are listed in Table 2.

**TABLE 2:** Algorithm equations.

No.	Input *x*	Algorithm equations	Output *y*
1	*x*_*1*_ *= T*_*fg.in*_	$y_{1}=\frac{2*LW_{1}}{1+e^{-2*\left(IW_{1}*x_{1}+a_{1}\right)}}-LW_{1}+b_{1}$	*y*_*1*_ *=* [*T*_*fg.in,*_ *φ*_*lean*_ *, y*_*CO2,*_ *C*]
2	*x*_*2*_ *= m*_*lean*_	$y_{2}=\frac{2*LW_{2}}{1+e^{-2*\left(IW_{2}*x_{2}+a_{2}\right)}}-LW_{2}+b_{2}$	*y*_*2*_ *=* [*φ*_*lean*_ *,C*]
3	*x*_*3*_ *= ln(L/G)*	$y_{3}=\frac{2*LW_{3}}{1+e^{-2*\left(IW_{3}*x_{3}+a_{3}\right)}}-LW_{3}+b_{3}$	*y*_*3*_ *=* [*T*_*fg.in,*_ *φ*_*lean*_ *, y*_*CO2,*_ *C,η*_*CO2*_]
4	*x*_*4*_ *= ln(Q/L)*	$y_{4}=\frac{2*LW_{4}}{1+e^{-2*\left(IW_{4}*x_{4}+a_{4}\right)}}-LW_{4}+b_{4}$	*y*_*4*_ *=* [*φ*_*lean*_ *, y*_*CO2,*_ *C,η*_*CO2*_]

### 3.3 Algorithm Steps

**FIGURE 3:**
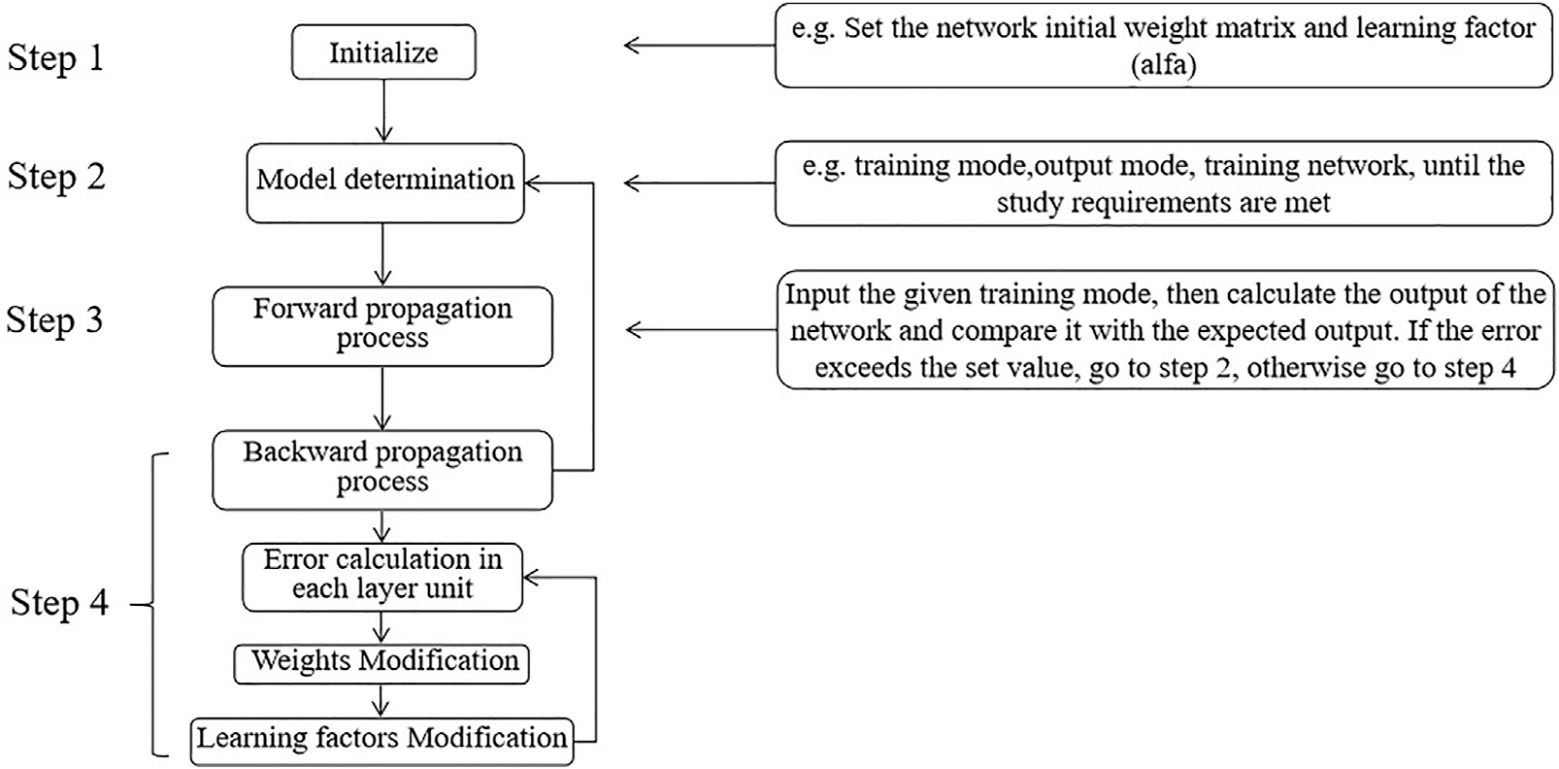
The steps of BPNN algorithm.

Based on the BPNN model in Figure 1, concerning a variety of algorithms in previous literature (Mudhasakul et al., 2013; Wang et al., 2017; Li et al., 2016), the algorithm steps are designed in Figure 3 (Talib and Hussin, 2017; Almeida and Leit, 2019).

In step 4, error calculation and the momentum term is introduced in this study. In other words, the last weight modification is taken into account in the current weight modification. At the same time, the modification of the weight of the previous unit is also included. In the BPNN used in this article, the weight modification from the hidden layer to the output layer is expressed in C++ language as:

delta_out= y[m]*(1-y[m])*(out_tech[m]-y[m]);
w_hid_out[j]+ = alfa*delta_out*h[j]+ beita*temp_hid[j]+ eita*temp hid[j-1];
temp_hid[j]= alfa*delta_out*h[j]+ beita*temp_hid[j];

Among them, w_hid_out[j] represents the weight from the jth unit of the hidden layer to the output unit; y[m] is the network output value of a certain training mode; and out_tech[m] is the corresponding target value; alfa represents the learning factor; beita is a constant of 0 or greater than 0, indicating the degree of inheritance of the last weight modification, which is called the inheritance factor; eita is a constant of 0 or greater.

In step 4, the learning factors are modified to prevent a too-slow convergence or oscillation or even divergence caused by the improper selection of the learning factor alfa. The method of changing the learning factor is adopted.

**FIGURE 4:**
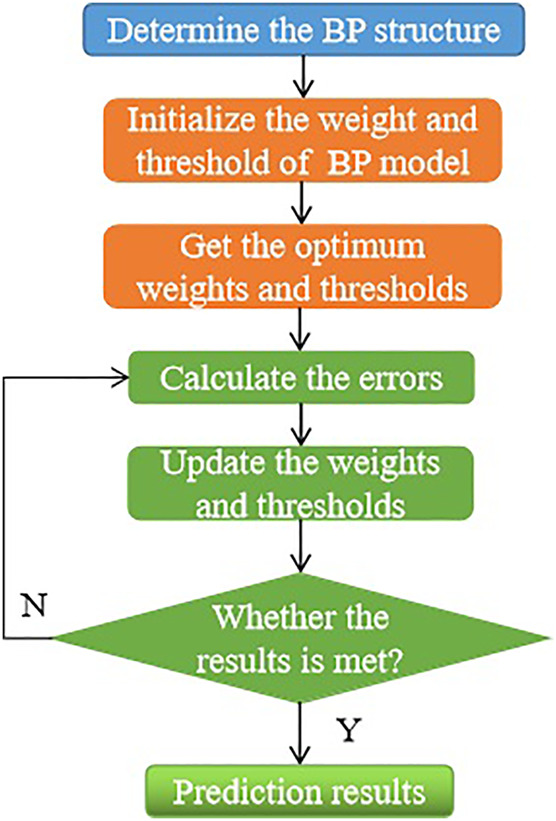
The concrete implementation flow of CO_2_ prediction based on the BPNN model.

Figure 4 summarizes and shows the concrete implementation flow in CO_2_ prediction based on the BPNN model (Sun et al., 2021).

## 4 Results and Discussion

The boiler operating parameters were downloaded from the onsite real-time PI database from the coal-fired power plant, and 115 groups of typical working conditions of the boiler during load-up, load-down, and load stabilization periods were selected. Among them, 80 groups were used as training samples, and 35 groups were used as test samples. Each set of samples includes the input value and expected output value of the neural network.

### 4.1 The Relationship Between Mean Squared Error and Training Sessions

**FIGURE 5:**
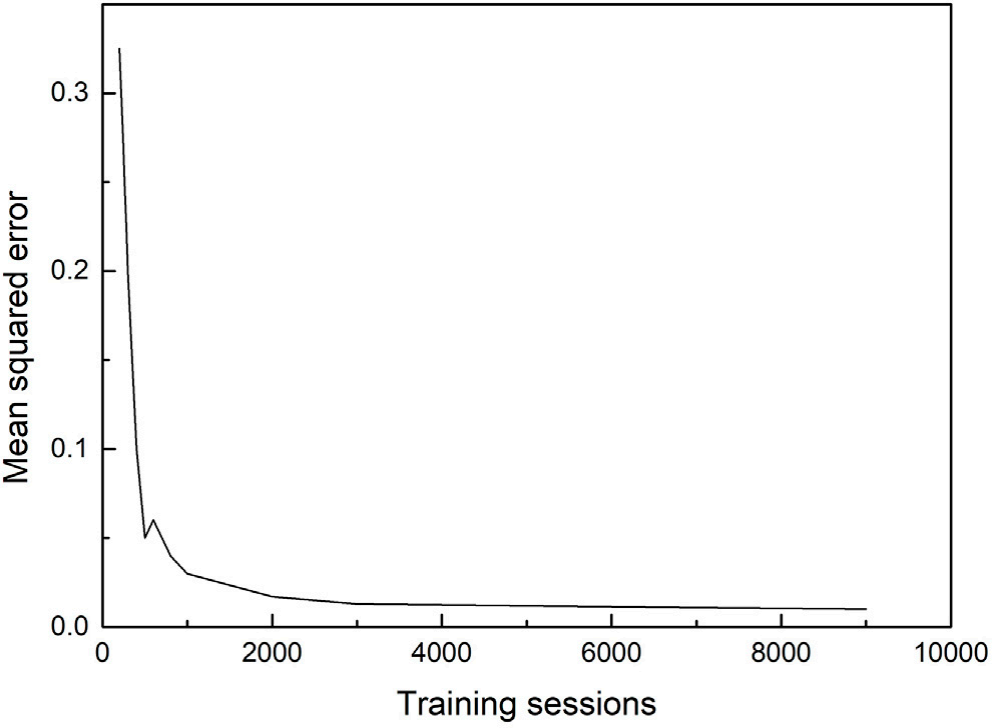
Mean squared error as a function of the number of training sessions.

Since the collected data contain noise, the artificial neural network will record all the data containing noise. If the training times are too many, it will not be able to output appropriate results and will not have good generalization ability. The performance of the sample data is mainly measured by its generalization ability. The stronger the generalization ability, the stronger the essential connection between the input and output in the sample data. Therefore, in this project, the training and testing are carried out alternately, that is, for each training time, the test is performed once, to roughly obtain the curve of the mean square error changing with the number of training times as shown in Figure 5.Here, the mean square error *D* is defined as

$$D=\sqrt{\sum_{p=1}^{m}\sum_{j=1}^{n}{\left(d_{pi}-y_{pi}\right)}^{2}/2mn,}$$ (6)

where *m* is the number of pattern pairs in the training sample, *n* is the number of network output layer units, *d*_*pi*_ is the expected output value of the network, and *y*_*pi*_ is the actual output value. When the number of training sessions is less than 2,000, the mean square error decreases rapidly with the increase of training sessions. When the number of training sessions is more than 2,000 times, the degree of change in the mean squared error does not change much with the increase in the number of training sessions.

### 4.2 Back Propagation Neural Network Modelling Evaluation

The neural network model has been developed considering the inputs as circulation amount of MEA, inlet flue gas temperature in adsorption tower, total flue gas flow, the ratio of liquid–gas, the average molecular weight of lean liquid, and the heat required for regeneration per unit of MEA, which are acquired from the experimental results to predict the output results as CO_2_ capture amount. The maximum experiment points were 60 in each section, and the percentage of experiments that were trained was between 10 and 20%. The forecasting ability of the model for the engine response in this study has shown a good agreement with the correlation statistics. However, the total uncertainty associated with model prediction is the consequence of different input aspects.

**FIGURE 6:**
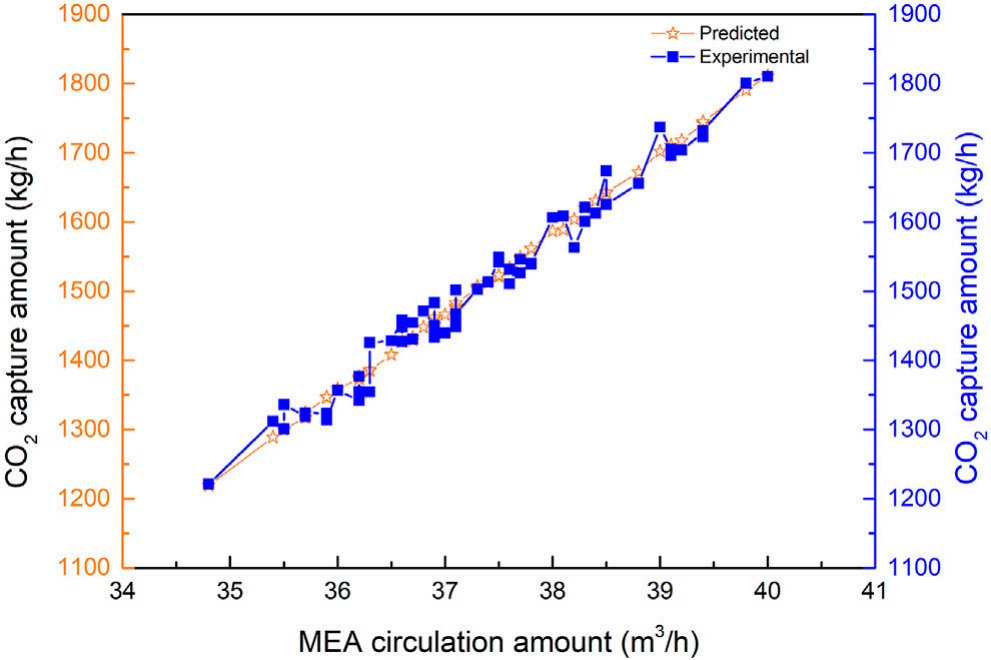
Comparison of CO_2_ capture predicted data and experimental data with the change of MEA circulation amount.

As observed from Figure 6 to Figure 13, the predicted values are commendably concurrent with the actual monitor for the experimental operation. The value of the CO_2_ capture amount was from 1,200 to 1,800 kg/h during the implementation of the experiments. The value of liquid temperature was between 40 and 70°C, and the gas temperature was between 20 and 75°C during the implementation of the experiments. All these parameters were related to the experiment variables. This implies that the robustness of the prediction model to estimate CO_2_ capture performance simultaneously with outstanding precision is irrespective of the case of the experimental operation.

#### 4.2.1 Sensitivity Analysis of the Monoethanolamine Circulation Amount

The comparison of the predicted values *vs*. the experimental values for CO_2_ capture is shown in Figure 6, with the change of circulation amount of MEA from 34 to 40 m^3^/h when the other parameters were stable. The predicted values exhibit an extremely low MSRE of 0.002372. Additionally, the predicted results are extremely consistent with the experimental results. The increasing MEA circulation amount causes the increase of CO_2_ capture amount during the experiment test. Also, the CO_2_ capture amount is sensitive to the MEA circulation amount.

**FIGURE 7:**
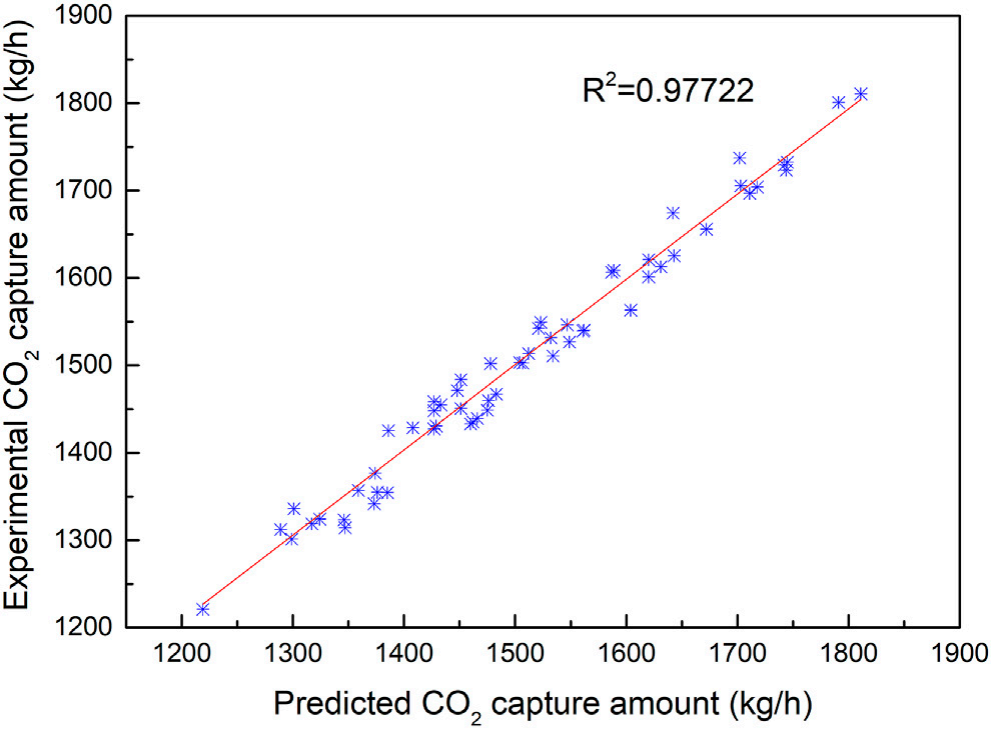
Fitting results of CO_2_ capture predicted data and experimental data with the change of MEA circulation amount.

Figure 6 illustrates the fitting results of the predicted values with the experimental results for CO_2_ capture amount by the BPNN model. From Figure 7, it can be concluded that the CO_2_ capture amount is highly dependent on the circulation amount of MEA. It reveals the value of MSRE content as 0.001945, correlation *R* ^2^ as 0.97722, and the CO_2_ capture accuracy of the prediction results of the BPNN algorithm to be as high as 96.6%.

#### 4.2.2 Sensitivity Analysis of the Total Flue Gas Flow

**FIGURE 8:**
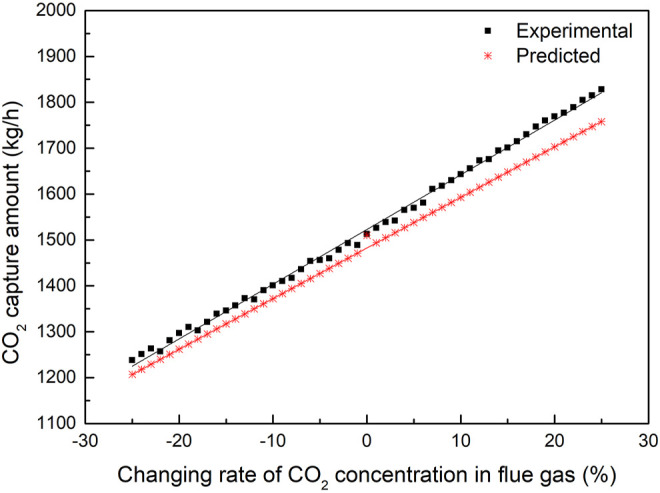
Comparison of CO_2_ capture predicted data and experimental data with the change of CO_2_ concentration.

**FIGURE 9:**
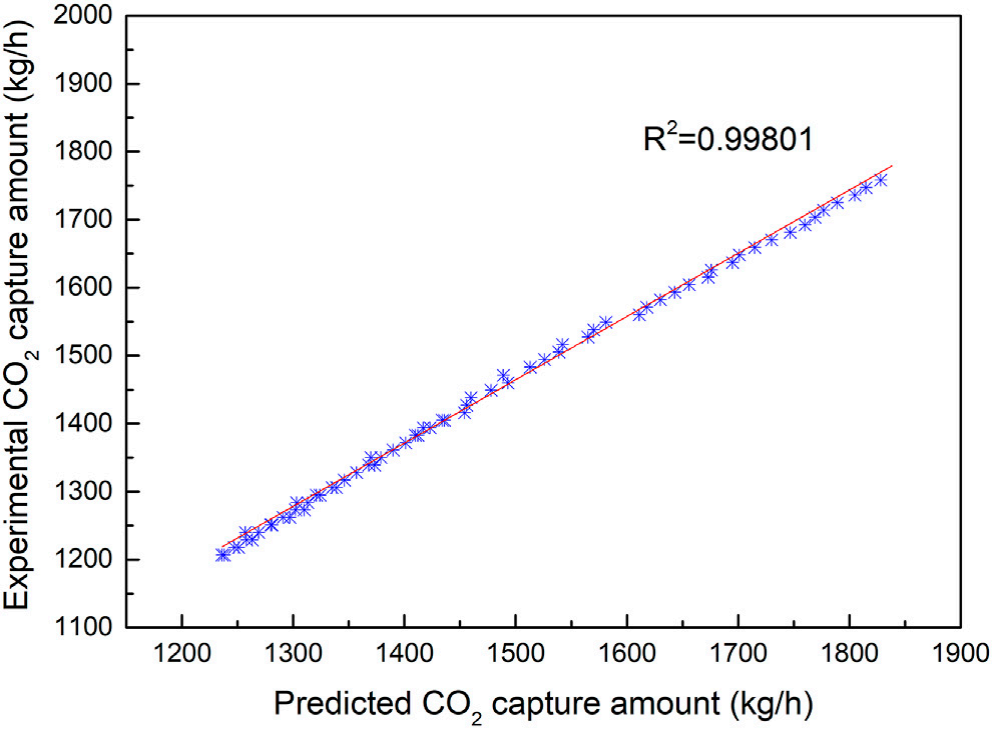
Fitting results of CO_2_ capture predicted data and experimental data with the change of CO_2_ concentration.

Figures 8, 9 reveal that the sensitivity of the CO_2_ capture amount changed with the flue gas flow when the other parameters were stable and the correlation between the predicted and experimental CO_2_ capture amount. The flue gas flow is related to the load of the boiler, and the content of CO_2_ in the flue gas increases with increasing load. The standard CO_2_ content is at the 75% boiler load, and the CO_2_ capture amount changing rate is based on the standard boiler load.

Figure 9 illustrates that the CO_2_ concentration in the flue gas increases, and the temperature of the flue gas exiting the absorption tower increases accordingly because the reaction heat will increase with the increase in carbon dioxide concentration, so the temperature of the flue gas exiting the absorption tower increases. The CO_2_ capture amount increases with the increase of the boiler load and flue gas flow. The heat required for unit regeneration of the absorbent increases accordingly because the load rate of the rich liquid will also increase correspondingly with the increase of carbon dioxide. When the load rate of the lean liquid remains unchanged, the energy required for the regeneration of the absorbent will also correspondingly increase because the higher the temperature is, the higher the regeneration efficiency will be.

When the loading rate of the lean liquid increases, the energy required for unit regeneration of the absorbent drops significantly because the loading rate of the lean liquid represents the effect of solvent regeneration. The increase in the amount of solvent required for absorbing unit flue gas is because a large load rate of lean liquid means that the solvent cannot be fully used for absorption, so more solvent is needed to absorb carbon dioxide in the flue gas. When the CO_2_ concentration in the flue gas increases, the rich loading increases. Since the regeneration energy remains unchanged, the CO_2_ that can be desorbed could also be constant, therefore the lean loading will increase.

When the capture rate increases and the load rate of the lean and rich liquid remains unchanged, to increase the capture rate of carbon dioxide, the total flow rate of the absorbent must be increased under the condition that the total flow rate of flue gas remains unchanged. When the absorbent flow rate increases, the gas–liquid (G/L) ratio decreases. Similarly, when the total heat required for the absorbent regeneration remains unchanged, the total flow rate of the absorbent must be increased, so when the capture rate increases, the heat required for unit regeneration of the absorbent will increase accordingly. The correlation between the experimental and predicted value *R* ^2^ is 0.99801.

Compared with the results of Figure 7, the correlation between the CO_2_ capture amount and the concentration of CO_2_ is better than that of MEA circulation influence on the CO_2_ capture amount, which proves that MEA circulation is more sensitive to the effect of CO_2_ absorption. The amount of CO_2_ capture is not very sensitive to the effect of different boiler load conditions compared with the sensitive effect of MEA circulation amount.

#### 4.2.3 Sensitivity Analysis of Steam Consumption

**FIGURE 10:**
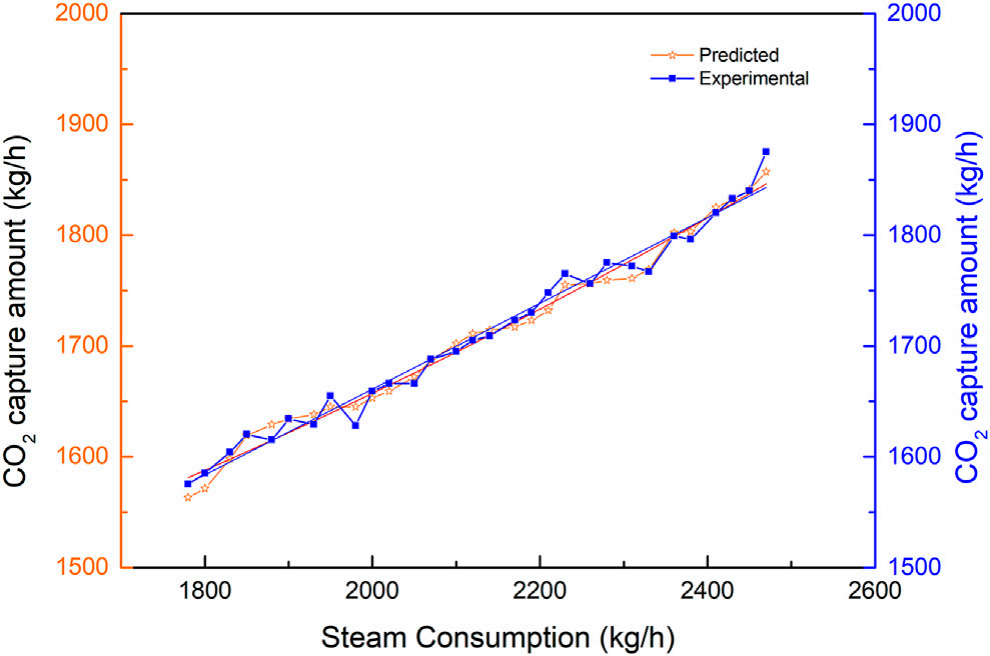
Comparison of CO_2_ capture predicted data and experimental data with the change of steam consumption.

**FIGURE 11:**
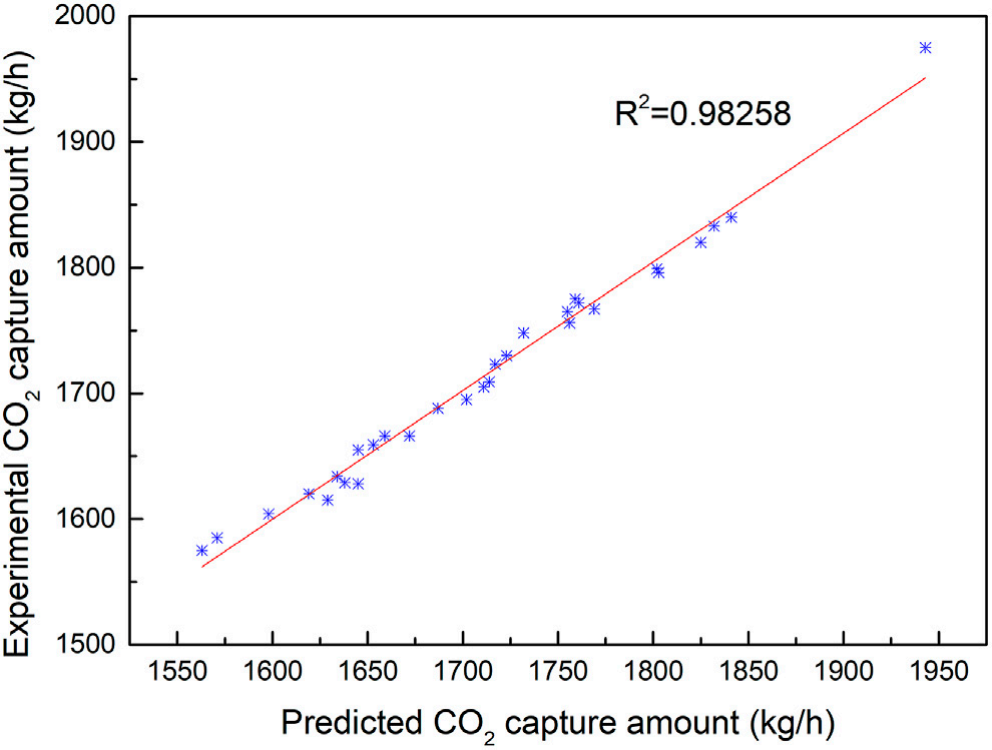
Fitting results of CO_2_ capture predicted data and experimental data with the change of steam consumption.

Figures 10, 11 reveal the sensitivity of the CO_2_ capture amount changed with steam consumption when the other parameters were stable and the correlation between the predicted and experimental CO_2_ capture amount. The steam consumption is related to the concentration of MEA solution. It is the main factor for the regeneration efficiency and is related to the CO_2_ absorption amount at the same time.

The comparison of the predicted values *vs*. experimental values for CO_2_ capture is shown in Figure 10, with the change in steam consumption when the other parameters were stable. The predicted values exhibit an extremely low MSRE of 0.001987. Additionally, the predicted results are extremely consistent with the experimental results. The change in steam consumption plays an important role in the CO_2_ capture amount during the experiment test. Figure 11 illustrates the fitting results of the predicted values with the experimental results for CO_2_ capture amount by the BPNN model. It can be concluded that the CO_2_ capture amount is dependent on steam consumption. The correlation between the experimental and predicted value *R* ^2^ is 0.98258.

#### 4.2.4 Sensitivity Analysis of the Absorption Temperature

**FIGURE 12:**
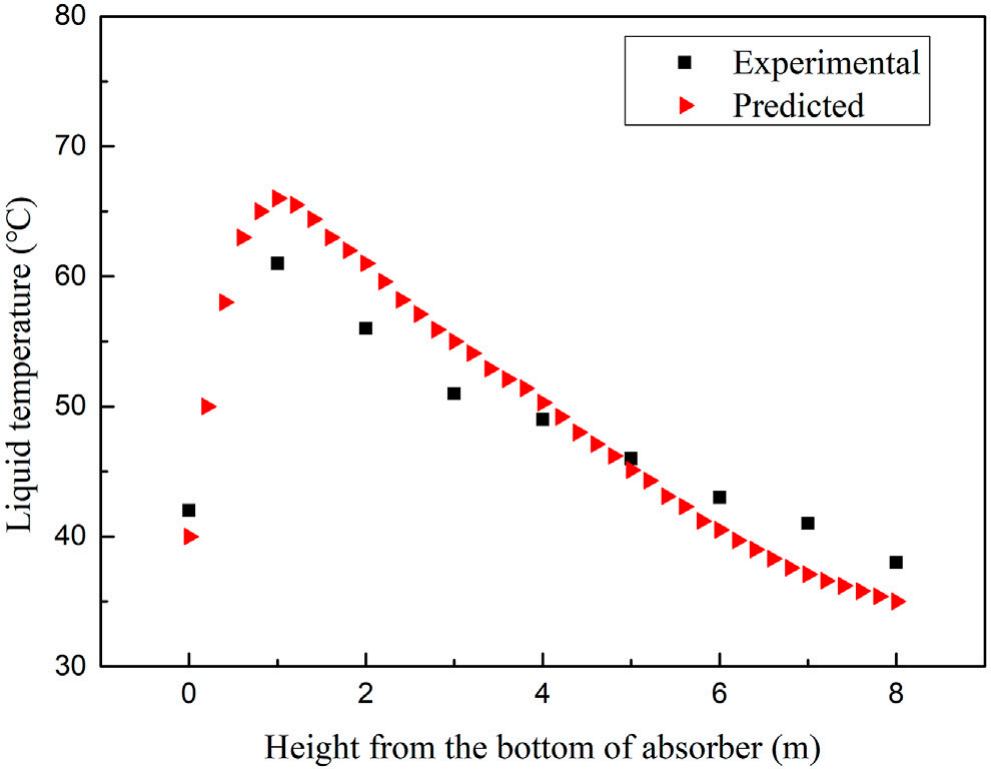
Sensitivity analysis of the liquid temperature along the packed column of the absorber.

Temperature is a critical parameter in the absorption and regeneration process for its importance on the CO_2_ capture amount and energy consumption during the MEA aqueous regeneration process. Various operation parameters such as the solubility of CO_2_ in an amine solution, transport parameters, kinetic reaction rates, and L/G ratio are all due to temperature. The absorbing column operates in a counter-current mode (Afkhamipour and Mofarahi, 2014), and rich CO_2_ flue gas is fed to the bottom of the absorber, while lean amine is fed to the top of the absorber. As CO_2_ is absorbed by the amine solution, the heat released from the reactions increases the temperature of the solution coming down from the top of the absorber. The flue gas at the bottom of the absorber takes up part of the heat evolved from the rich amine. Hence, the flue gas temperature increases from the bottom upward to the near top of the absorber, where the lean amine is heated by contact with the up-flowing flue gas. Because of the heat of the reaction, water is vaporized and is then condensed by the colder lean amine at the top of the absorber. When the temperature of the flue gas entering the absorption tower increases, the amount of solvent required to absorb a unit volume of flue gas increases rapidly because the absorption reaction is exothermic. When the temperature increases, the CO_2_ solubility decreases, thus the CO_2_ concentration in the liquid phase decreases. It is required to add more solvent to absorb more CO_2_. In Figure 12, the flue gas temperature of the absorption tower increases accordingly because when the reaction heat is constant, the inlet temperature is increased, and the outlet temperature also increases accordingly.

**FIGURE 13:**
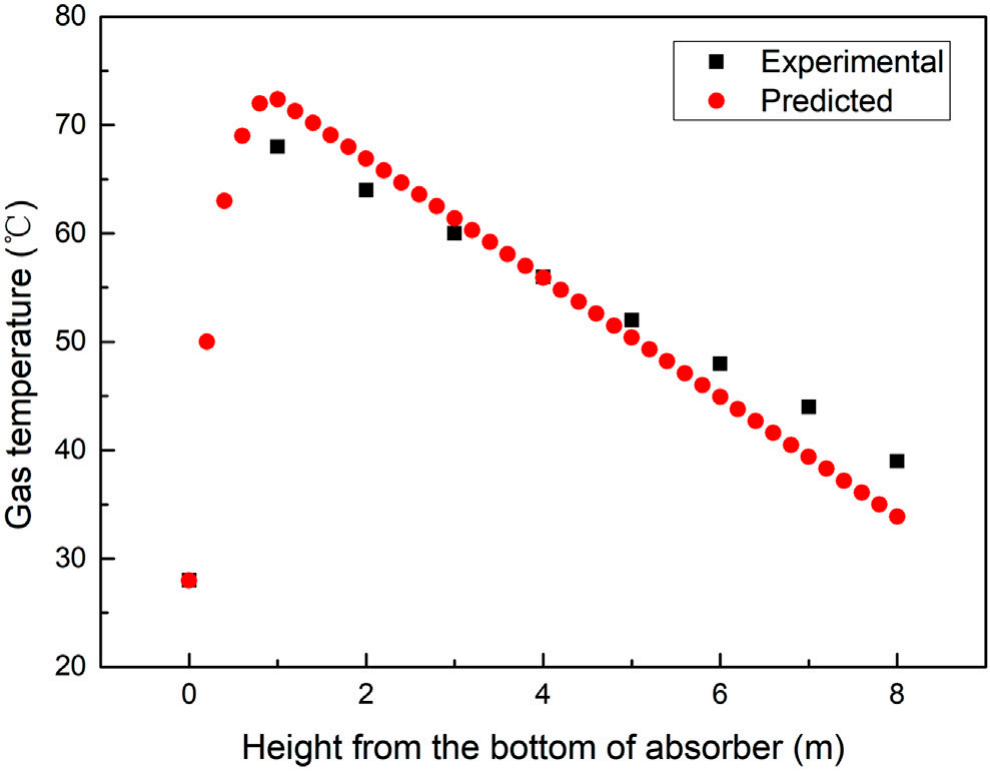
Sensitivity analysis of the gas temperature along the packed column of the absorber.

A noticeable temperature increase can be seen in the temperature profiles of the absorber as shown in Figures 12, 13. Due to the high L/G ratio in the operation, the location of the temperature increase was captured reasonably well in all the cases. The comparisons between the calculated results of temperature profiles and experimental data under the operating conditions are given in Table 1. The shapes of the temperature profiles predicted by the rate-based model using different mass transfer correlations and kinetic models vary considerably.

The increase of the temperature bulge typically increases the kinetics of CO_2_ absorption, but it will disrupt the vapor–liquid equilibrium (Versteeg et al., 1996). At high temperatures, the CO_2_ equilibrium partial pressure may begin to approach the CO_2_ partial pressure in the bulk gas. These conditions create a lack of a driving force, which results in a “pinch” where additional CO_2_ is not absorbed by the amine solution. As a result, the mass transfer performance of the column is reduced. In Figure 13, the temperature profiles show the typical behavior of the reactive absorption column, where a temperature bulge can be seen at the bottom of the column due to the high L/G ratio of the operation and simulation.

In general, the accuracy parameters chosen in the BPNN model cause discrepancies between the prediction profiles and experimental data. Therefore, the use of accuracy parameters in BPNN model in CO_2_ sensitivity analysis has greater confidence in the accuracy of the prediction model.

## 5 Conclusion

In this article, the BPNN algorithm is applied to the prediction of CO_2_ capture of amine solvent washing technology running in coal-fired power plants. The developed BPNN contains an input layer with 6 neurons, a single hidden layer with 10 neurons, and an output layer with 2 neurons. The prediction characteristic with interpolation and extrapolation along with the robustness of the model has been evaluated on a statistical platform containing different errors and performance analysis. The error analysis revealed that the developed model predicted the experimental results with a very high degree of accuracy with the value of MSRE ranging from 0.001945 to 0.002372 when the circulation amount of MEA changed. Sensitivity analysis suggests that under the given conditions, the flue gas amount has a marginal effect on the system performance, while further attention should be paid to the MEA circulation amount, which is crucial to the CO_2_ capture amount. The temperature profiles show the typical behavior of the reactive absorption column where a temperature bulge can be seen at the bottom of the column due to the high L/G ratio of the experimental and prediction results. The coefficient of correlation *R* ^2^ with the change of MEA circulation amount, change of CO_2_ concentration, and steam consumption are 0.97722, 0.99801, and 0.98258, respectively. It also proves that the parameter of MEA circulation is the most sensitive factor among the input parameters for CO_2_ capture. The experimental results show that the accuracy of prediction results of the BPNN algorithm is as high as 96.6%, which is better than several existing prediction methods. It shows that the BPNN algorithm is a consistent, reliable, and robust system identification tool for CO_2_ capture by the amine solvent scrubbing technique of operation in coal-fired power plants.

## Data Availability

The original contributions presented in the study are included in the article/Supplementary Material. Further inquiries can be directed to the corresponding author.

## Nomenclature

*a*: the threshold from the input layer to hidden layer
*b*: the threshold from the hidden layer to output layer
***d*** **_*pi*_**: the expected output value of the network
***y*** **_*pi*_**: the actual output value
*C*: MEA concentration, kmol/m^3^
***C*** **_*s*_**: steam consumption in regeneration tower
*G*: total flue gas flow rate, m^3^/h
*L*: speed of MEA, kmole/hr
*L/G*: the ratio of liquid–gas
*m*_*lean*_: average molecular weight of the lean liquid
*n*: the numbers of samplings
*o*_*i*_: the predicted value
*Q*: total heat required for MEA regeneration, GJ/hr
*Q/L*: heat required for regeneration per unit of MEA, GJ/kmole
*T*_*fg.in*_: inlet flue gas temperature in adsorption tower, K
*T*_*fg.out*_: outlet flue gas temperature in adsorption tower, K
*t*_*i*_: the observed value
*W*: weight vectors
*x*: the input in the neural network
*y*: the output in the neural network
*y*_*CO2*_: amount of carbon dioxide absorbed per unit of MEA, mole/%

### Greek Symbol

*φ*_*lean*_: lean liquid loading rate
*η*_*CO2*_: CO_2_ capture efficiency, %

### Abbreviations

BPNN: back propagation neural network
MSRE: mean square root error
MEA: monoethanolamine
